# Linked-Read Whole Genome Sequencing Solves a Double *DMD* Gene Rearrangement

**DOI:** 10.3390/genes12020133

**Published:** 2021-01-21

**Authors:** Maria Elena Onore, Annalaura Torella, Francesco Musacchia, Paola D’Ambrosio, Mariateresa Zanobio, Francesca Del Vecchio Blanco, Giulio Piluso, Vincenzo Nigro

**Affiliations:** 1 UOSID Genetica Medica e Cardiomiologia, Dipartimento di Medicina di Precisione, Università degli Studi della Campania “Luigi Vanvitelli”, 80138 Napoli, Italy; maria.elena.onore@gmail.com (M.E.O.); annalaura.torella@unicampania.it (A.T.); paola.dambrosioMD@gmail.com (P.D.); mt.zanobio@gmail.com (M.Z.); francesca.delvecchioblanco@unicampania.it (F.D.V.B.); giulio.piluso@unicampania.it (G.P.); 2 Telethon Institute of Genetics and Medicine, 80078 Pozzuoli, Italy; francescomusacchia@gmail.com

**Keywords:** 10× Genomics, linked-read WGS, undiagnosed diseases, DMD gene, muscular dystrophy

## Abstract

Next generation sequencing (NGS) has changed our approach to diagnosis of genetic disorders. Nowadays, the most comprehensive application of NGS is whole genome sequencing (WGS) that is able to detect virtually all DNA variations. However, even after accurate WGS, many genetic conditions remain unsolved. This may be due to the current NGS protocols, based on DNA fragmentation and short reads. To overcome these limitations, we applied a linked-read sequencing technology that combines single-molecule barcoding with short-read WGS. We were able to assemble haplotypes and distinguish between alleles along the genome. As an exemplary case, we studied the case of a female carrier of X-linked muscular dystrophy with an unsolved genetic status. A deletion of exons 16–29 in *DMD* gene was responsible for the disease in her family, but she showed a normal dosage of these exons by Multiplex Ligation-dependent Probe Amplification (MLPA) and array CGH. This situation is usually considered compatible with a “non-carrier” status. Unexpectedly, the girl also showed an increased dosage of flanking exons 1–15 and 30–34. Using linked-read WGS, we were able to distinguish between the two X chromosomes. In the first allele, we found the 16–29 deletion, while the second allele showed a 1–34 duplication: in both cases, linked-read WGS correctly mapped the borders at single-nucleotide resolution. This duplication in trans apparently restored the normal dosage of exons 16–29 seen by quantitative assays. This had a dramatic impact in genetic counselling, by converting a non-carrier into a double carrier status prediction. We conclude that linked-read WGS should be considered as a valuable option to improve our understanding of unsolved genetic conditions.

## 1. Introduction

Duchenne Muscular Dystrophy (DMD) and Becker Muscular Dystrophy (BMD) are X-linked recessive neuromuscular disorders due to mutations in the dystrophin gene (MIM 300377). Whereas deletions or duplications of one or more exons in *DMD* gene account for about 75% of cases, small mutations (missense, nonsense, and splice site variations), short insertions/deletions of bases, and small inversion are detected in the remaining 20–25% of cases [1,2,3]. Moreover, about 1–2% of cases show elusive rearrangements or deep intronic variants [4,5] Therefore, the initial genetic test searches deletions or duplications by quantitative methods. PCR-based techniques, such as Log-PCR [6] and Multiplex Ligation-dependent Probe Amplification (MLPA) [7], or customized array comparative genomic hybridization (Array-CGH), have been used [8]. Next generation sequencing (NGS) is then used to detect the remaining mutations [9,10].

The updated care considerations for DMD suggest NGS after MLPA in the diagnostic pipeline [11], but the technologies are moving very quickly and we cannot exclude that the two-step protocol could be converted into a single NGS-based test [12].

At present, most laboratories are using robust NGS gene panels or Whole Exome Sequencing (WES) and more recently Whole Genome Sequencing (WGS). Despite great expectations, even the WGS may prove insufficient. This may be due to several causes, such as elusive intronic defects, but also complex structural variants that require further testing.

Nowadays, a complete analysis of the genome is not possible because the Short-Read sequencing technologies provide information to call Single Nucleotide Variants (SNVs) but fail at long-range haplotypes construction [13]. Because large regions of the genome consist of a high number of repeated sequences, the current analytical methods are unable to call large structural variants (SVs) such as insertions, deletions, duplications, and rearrangements of genomic segments greater than 50 bp [14]. Recently, Long-Read technologies emerged to be effective for the detection of SVs [14,15] and SNVs [16].

The 10× Linked-Read sequencing strategy combines the performances of Short-Read sequencing with the long-range information. The addition of barcode information provides better alignments in regions of the genome typically inaccessible than standard Short-Read approaches [17]. The determination of long-range haplotypes is the first advantage of Linked-Read sequencing [18]. In the present study, we applied the linked-read WGS to solve a complex genetic status with important reproductive implications.

## 2. Materials and Methods

### 2.1. Index Case

The index case is a 6-year-old. Following an episode of myoglobinuria, after fever treated with ibuprofen, he was admitted to the emergency ward and underwent laboratory evaluation. CK levels were very high (143,000 U/L) and progressively dropped after NaCl infusion and rest (42,600 U/L). He was then admitted to the pediatric ward and a genetic evaluation was requested in the suspicion of a neuromuscular disease. His family history, previous medical history and developmental milestones were normal. Indices of inflammation, plasma acylcarnitine profile, electrocardiogram (ECG), echocardiogram and neurological examination were normal. He did not present Gower sign, but he just had difficulty to stand on his heels. The persistently elevated CK levels, ranging from 303 to 1902 U/L at rest and negative metabolic profile led us to test for mutations in the *DMD* gene. His mother, grandmother (163 U/L) and two maternal aunts (48 U/L, 38 U/L) were asymptomatic with mildly elevated or normal CK levels. Genetic counselling suggested molecular analysis in the proband and female family members to evaluate carrier status.

### 2.2. Sample Collection

Genomic DNA from the proband and his family members enrolled for the study was isolated from peripheral blood using FlexiGene DNA Kit (Qiagen, Hilden, Germany), according to the manufacturer’s instruction. Written informed consent was obtained from all family members participating in the study.

### 2.3. Multiplex Ligation-Dependent Probe Amplification (MLPA)

Multiplex ligation-dependent probe amplification (MLPA) assay was performed with the SALSA MLPA P034/P035 DMD kit (MRC Holland, Amsterdam, The Netherlands), according to the manufacturer’s recommendations. The SALSA MLPA P095 Aneuploidy kit (MRC Holland, Amsterdam, The Netherlands) was used in patient II-3 to exclude the presence of an extra X-chromosome.

Following denaturation of Genomic DNA, MLPA mix and the probes are briefly added to the samples for overnight hybridization. Ligation reaction and multiplex PCR reaction were performed with a Ligase-65 master mix and polymerase master mix, respectively. The PCR products were then separated on an ABI 3130xL automatic DNA sequencer (Life Technologies, Carlsbad, CA, USA). Coffalyser.Net package (MRC-Holland, Amsterdam, The Netherlands) was performed for MLPA data analysis.

### 2.4. Array CGH and CGH+SNP

Labeling, hybridization and data analysis were performed according to the manufacturer’s specifications and as previously reported [19,20]. The Motor Chip, a customized array CGH for Neuromuscular disorders [19], was used to analyze the male patient (III-1), his mother (II-1) and the maternal aunt with an ambiguous genetic status of potential BMD carrier (II-3). SurePrint G3 ISCA v2 CGH+SNP 4×180K microarray was also used to analyze the maternal aunt (II-3). Following hybridization and microarray scanning, data analysis was carried out using the Cytogenomics software (Agilent Technologies, Santa Clara, CA, USA).

### 2.5. 10× Genomics Whole Genome Sequencing (10× WGS)

10× Genomics WGS was performed on the maternal aunt (II-3). High Molecular Weight (HMW) genomic DNA was extracted from 200 μL fresh peripheral blood sample using the MagAttract^®^ HMW DNA Kit (Qiagen, Hilden, Germany) according to the manufacturer’s recommendations. Genomic DNA was quantified with Qubit dsDNA HS Assay Kit.(Thermo Fisher Scientific, Waltham, MA, USA) An emulsion system with a microfluidic chip to partition and barcode high-molecular weight (HMW) DNA molecules was used by this technology. 

A library of Genome Gel Beads was combined with 2.5 ng of HMW gDNA in Master mix and partitioning oil to create Gel Bead-In-EMulsions (GEMs) on a Chromium Genome Chip. The assembled Chip was loaded in the Chromium Controller. After the run, GEMs were transferred in a PCR 8-tube strip and exposed to isothermal incubation to produce barcoded fragments; in this way, all fragments that originate from a small number (5 to 10) of DNA molecules 50 Kb in length are labelled with a common 10× Barcode. 

Solid phase reversible immobilization (SPRI) beads were used for DNA size selection. Read 1 sequence and the 10× barcode were added to the molecules during the GEM incubation, whereas P5 and P7 primers, Read 2 and Sample Index are added during library construction (via end repair, A-tailing, adaptor ligation, and amplification). Fragment library size was determined using Agilent Bioanalyzer High Sensitivity DNA chip and DNA 1000 chip, respectively. Sequencing was performed using an Illumina Novaseq 6000 System and standard Illumina sequencing primers. Genome libraries were run using paired-end sequencing with single indexing. 

### 2.6. 1–10× Data Analysis and Visualization

Long Ranger software was used for analyzing the 10× sequencing data with the “WGS” pipeline and default settings. The Lariat aligner mapped the Linked-Reads to the reference Human genome sequence, while GATK with Long Ranger pipeline generated calls of single nucleotide and small indel variants [21]. After variant calling, a phasing method optimized for 10× barcode is used to phase the identified SNPs [22]. In our Chromium run, the number of GEMs collected was 1,538,258 and the corrected estimated DNA loaded was 2.70 ng. A total of 376,532,512 reads, with a median fragment length of 335 bp, was generated. Only 98% (369,001,861.76) of the reads were mapped to the reference genome, while 97.4% of the identified heterozygous SNPs were correctly phased. We called 35 large SVs and 5404 short deletions with an average sequencing depth of 16.8×. 

The output of Long Ranger pipeline was analyzed with the Loupe software (Version 2.1.1) to visualize large SVs, inter-chromosomal translocations, gene fusions, as well as inversions or deletions. Loupe viewer also displayed the analyzed genome as two haplotypes, excluding unphased molecules visualized below.

Two different manners are used to visualize the Linked-Reads: Haplotype discrimination and Lariat mode. In the first one, the two haplotypes and the unphased molecules are marked with different colors while in second one, the reads of different colors are linked depending on their alignment properties.

## 3. Results

**Figure 1:**
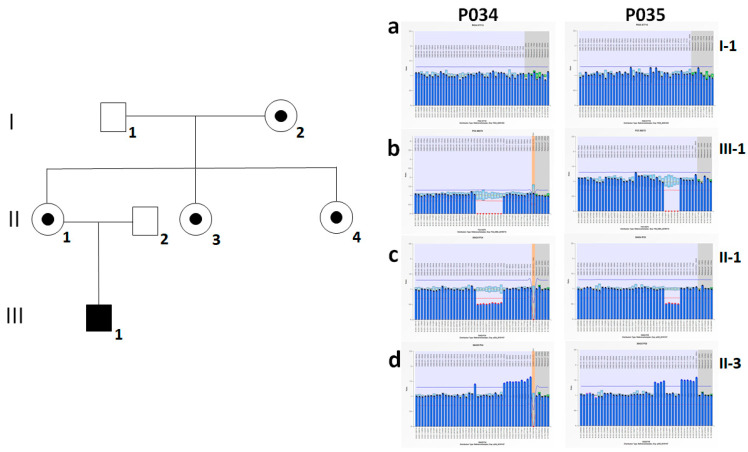
Family pedigree and Results of Multiplex Ligation-dependent Probe Amplification (MLPA) analysis. In family pedigree (on the left) a black box indicates the affected male, while a black dot in the circle indicates carrier females. Results of MLPA analysis for P034 and P035 SALSA DMD kits are presented as bar graphs (on the right) corresponding to (**a**) the maternal grandfather (I-1), (**b**) the affected male (III-1), (**c**) his heterozygous carrier mother (II-1), and (**d**) the carrier maternal aunt (II-3) showing the unexpected complex rearrangement.

We report the case of a 6-year-old with an elevated CK and a diagnosis of muscular dystrophy. MLPA analysis of *DMD* gene identified a deletion of exons 16–29 [NM_004006.2: c.(1812+1_1813–1) (4071+1_4072–1)del] in hemizygosis in the affected male (III-1, Figure 1b), and in heterozygosis in his carrier mother (II-1, Figure 1c), in the maternal grandmother (I-2, data not shown), as well as in a maternal aunt (II-4, data not shown). The other maternal aunt (II-3), aged 32, showed a normal dosage of exons 16–29, compatible with a “non-carrier” status (Figure 1d). Unexpectedly, we calculated a triple dosage of exons 1–15 and exons 30–34. To investigate for a possible Triple X syndrome, SALSA MLPA P095 Aneuploidy kit was also used in II-3 and this condition was excluded (data not shown). Our hypothesis was a more complex rearrangement that involved the flanking regions of the *DMD* deletion identified in the affected male (III-1). 

**Figure 2:**
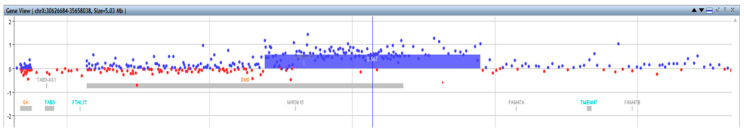
Array CGH + SNP analysis. Blue and red dots correspond to Log_2_ positive or negative ratio values (Y-axis) for each probe present in this specific chromosome interval (X-axis). Array CGH + SNP profile of maternal aunt (II-3): a duplication of about 1.51 Mb was detected with 133 probes (highlighted with a blue box). The duplicated region includes the first 34 exons of the *DMD* gene but also the 0.5Mb 5′ region.

To confirm and further define the possible deletion/duplication events we used a 180K array CGH + SNP analysis. We identified in II-3 a wider duplication of about 1.51 MB at Xp21.1 (minimum duplicated region chrX:32387659–33897065, detected with 133 probes). The duplicated region not only contained the first 34 exons of *DMD* but also a >500-kb upstream region (Figure 2). 

**Figure 3:**
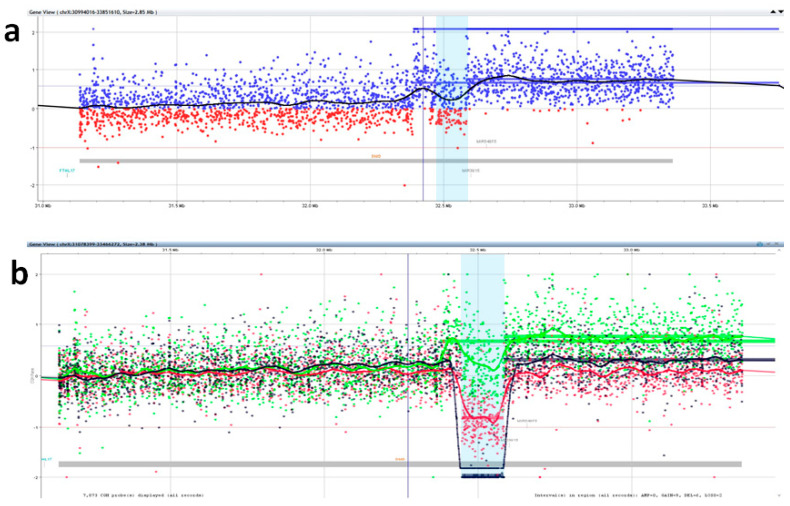
MotorChip Analysis. Blue and red dots correspond to Log2 positive or negative ratio values (Y-axis) for each probe present in this specific chromosome interval (X-axis). The light blue bar corresponds to the 16–29 exons of DMD. CGH panels showing the Log2 ratio profile of probes as a colored line: (**a**) complex duplication in the maternal aunt (II-3, black line); (**b**) the affected male (III-1, black line), his carrier mother (II-1, red line) and his maternal aunt (II-3, green line).

To further investigate the segregation of the aberrant X-chromosome in family members, we then used Motor Chip, a customized array CGH with a high probe density in *DMD* [23]. By comparing results from the affected male (III-1), his carrier mother (II-1) and the maternal aunt (II-3), we confirmed the wide and complex duplication in II-3 (Figure 3a) that included the duplicated region chrX:32385323–33757175, detected with 1126 probes, in which the normal copy number for exons 16–29, corresponding to the region chrX:32447837–32584015 and detected with 211 probes, was perfectly coincident with the deletion in the affected patient and his carrier mother (Figure 3b). 

However, these results did not clarify whether the non-contiguous duplication of exons 1–15 and 30–34 with normal dosage of exons 16–29 observed in II-3 resulted from an unusual rearrangement in cis or from a duplication with/without translocation independent from the mutated X chromosome. 

To explain this point and to define the correct localization and orientation of the rearrangement, we used Linked-Read WGS by 10× Genomics to phase both alleles and to measure the structural aberration. 

**Figure 4:**
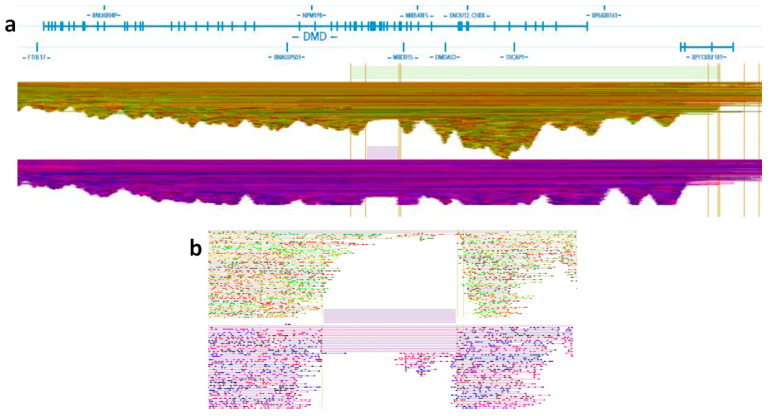
A selection of Linked-Reads in the *DMD* gene. Each dot corresponds to a single read; reads from the same GEM are horizontally linked with a line. The analyzed genome is displayed as two haplotypes: one placed on the top and the second one on the bottom. Linked-Read panels showing: (**a**) the duplication of *DMD* exons 1–34 with the 0.5 Mb-long upstream region is highlighted with a light green bar on the top haplotype, in which there are more phased reads; the deletion of *DMD* exons 16–29 is highlighted with a light purple bar on the bottom haplotype. The breakpoint-containing region is magnified in panel (**b**).

The highest quality score in the SV call was obtained for a tandem duplication of 1,520,188 bp on X-chromosome (ChrX: 32385204—33905872) that not only involved *DMD* exons 1–34 but also its the upstream region (ChrX: 33229695—33905872) for 676,177 bp, in which only a long intergenic non-coding RNA resulted annotated (RP11–305F18.1; ENSG00000233928) (Figure 4a). In addition, the list of SV call also included a deleted region of 137 kb (ChrX: 32447299—32583963), corresponding to the exons 16–29 of *DMD* (Figure 4b). The corresponding structural variants (SVs) are displayed in the Matrix View (Supplementary Figure S1).

Since the deletion was maternally inherited, the allele with the tandem duplication should be paternally inherited. The deletion is shown also in the Linked-Read view section (Figure 4b). 

The Loupe visualization displays additional unphased molecules, probably because this is a complex region of the genome. This could justify the presence of this SV in candidate list and its low associated score. To investigate the origin of *DMD* duplication that involves the paternal allele, MLPA analysis was also performed in the healthy maternal grandfather (Figure 1a). As expected from the phenotype, we showed a normal dosage of the *DMD* exons, thereby suggesting that the duplication originated in the male germline.

In summary, using 10× Linked-Read WGS, both alleles were phased and a complex *DMD* gene mutation was revealed. In summary, we detected a 137-kb deletion on maternal allele and a de novo 1.52-Mb duplication on the paternal allele. Notably, the larger duplication of the second allele apparently restores the normal dosage of exons 16–29.

## 4. Discussion

Although WGS is predicted to become a single-entry test for most genetic conditions, genome analysis and interpretation remains challenging. Genome sequence assembly, structural variation detection and haplotyping are the main difficulties [24]. Genomic alterations such as Single Nucleotide Variant (SNV), Insertion and Deletion (INDEL) and large Copy Number Variation (CNV) are detectable with WGS, but the identification of complex structural variants is quite impossible (SVs) [21], especially in repetitive regions of the genome [25].

Alternative sequencing technologies have been recently introduced with two different approaches: real-time sequencing and synthetic long reads [26]. Single-molecule-Real-Time (SMRT) sequencing from Pacific Biosciences (PacBio) and Nanopore Sequencing (ONT) are suited to SV detection, but the high cost and per-base error rate limited their application [27,28]. Alternatively, the Synthetic approaches provide the use of barcodes and the Illumina Synthetic Long-Read Sequencing platform, in which the DNA fragments are partitioned into a microtiter plate. The first limitation of this approach is the low DNA fragment coverage [29].

10× Genomics developed an alternative technology called Linked-Read Sequencing, in which the Short-Read Sequencing and the Long-Range information are combined. Linked-Reads are short sequencing reads with a unique 10× Barcode. The short-reads are randomly distributed along HMW DNA molecules [30]. With a microfluidic device, long DNA fragments are partitioned and barcoded in Gel-Bead-in-Emulsion (GEMs). The fragments originated from the same GEM are labelled with a common 10x Barcode. After the sequencing run, reads are aligned to the reference genome and linked together using the barcodes. The Long Ranger is the official software used in 10× Linked-Read Sequencing and provides allele phasing and haplotype reconstruction to detect large balanced and unbalanced SVs and small variants [31].

In this study, we have explored the utility of linked-read WGS for accurate molecular diagnosis and genetic counselling. In comparison with conventional WGS [20], this tool retains the reciprocal positions of sequenced fragments within each chromosome. This permits us to recognize the variations of each allele and to solve large regions of sequence similarity, such as duplications, gene conversions or the presence of pseudogenes.

As an exemplary case, we studied a family composed of women of reproductive age who are genetically linked with a 6-year-old boy affected by an X-linked muscular dystrophy. The in-frame deletion type suggested a BMD phenotype but the very elevated serum CK values and the early walking problems need to be still cautious in the BMD/DMD classification. An ambiguous reproductive risk of a 32-year woman was not clarified by MLPA and array CGH, so we decided to test the power of linked-read WGS.

We confirmed the deletion of exons 16–29 on the maternal allele plus a duplication of exons 1–34 on the paternal allele, which restores the normal dosage of exons 16–29. By 10× data analysis, this duplication involves a region of 1.52 Mb and encompasses not only the *DMD* gene, but also the 5′ upstream region.

Since the woman is a carrier of *DMD* exons 16–29 deletion, the transmission of the deleted allele to a male child will result in muscular dystrophy, while the transmission of the duplicated allele can also be pathogenic. It is difficult to predict the effect of the duplication of exons 1–34, because in theory a normal copy of the *DMD* gene is not interrupted by the duplication at the 5′. However, the healthy status of this carrier on the dystrophin protein should not be overstated, considering that mutations at 5′ of the gene may result in cardiomyopathies [32], and in this case there may be a compensative effect of the hypomorphic maternal allele. The absence of this mutation in the father cannot exclude a muscular dystrophy in hemizygosis.

## 5. Conclusions

In this study, we demonstrated that 10× Linked-Read WGS has the potential to detect large balanced/unbalanced SVs, such as the complex *DMD* gene mutation we revealed. The identification of these rearrangements is a common limitation of all technologies currently available, including Short-Read and Long-Read sequencing. The alternative approach, produced by 10× Genomics, combines a novel barcoding strategy of Short-Reads and Long-Range information and does not require the purchase of another NGS apparatus [21]. We conclude that linked-read WGS should be considered the correct choice to improve our understanding of unsolved genetic conditions in a very feasible way.

## Data Availability

Data is contained within the article or supplementary material.

## Supplementary Materials

The following are available online at https://www.mdpi.com/2073-4425/12/2/133/s1, Figure S1: Structural variant view.
