# Supplementary material for: Linked-Read Whole Genome Sequencing Solves a Double DMD Gene Rearrangement
Source: Genes (Basel). 2021 Jan 21;12(2):133. doi: 10.3390/genes12020133 (PMC7909759; doi:10.3390/genes12020133)

Supplementary Figure S1. Structural Variant View. In the Matrix view, the red box corresponds to the region of complex rearrangement; the tandem duplication of exons 1-34 and the overlapping barcodes can be visualized in green boxes while the overlapping barcodes corresponding to the deletion of exons 16-29 are shown in blue box. Dots represent overlapping barcodes. The two axes of the matrix represent two alleles and each dot (on the matrix graph) corresponds to a pair of loci. The diagonal line represents the expected barcode overlap between the two loci and the intensity of the colour represents the number of distinct common barcodes. The vertical green bars outside the matrix shows the average coverage.

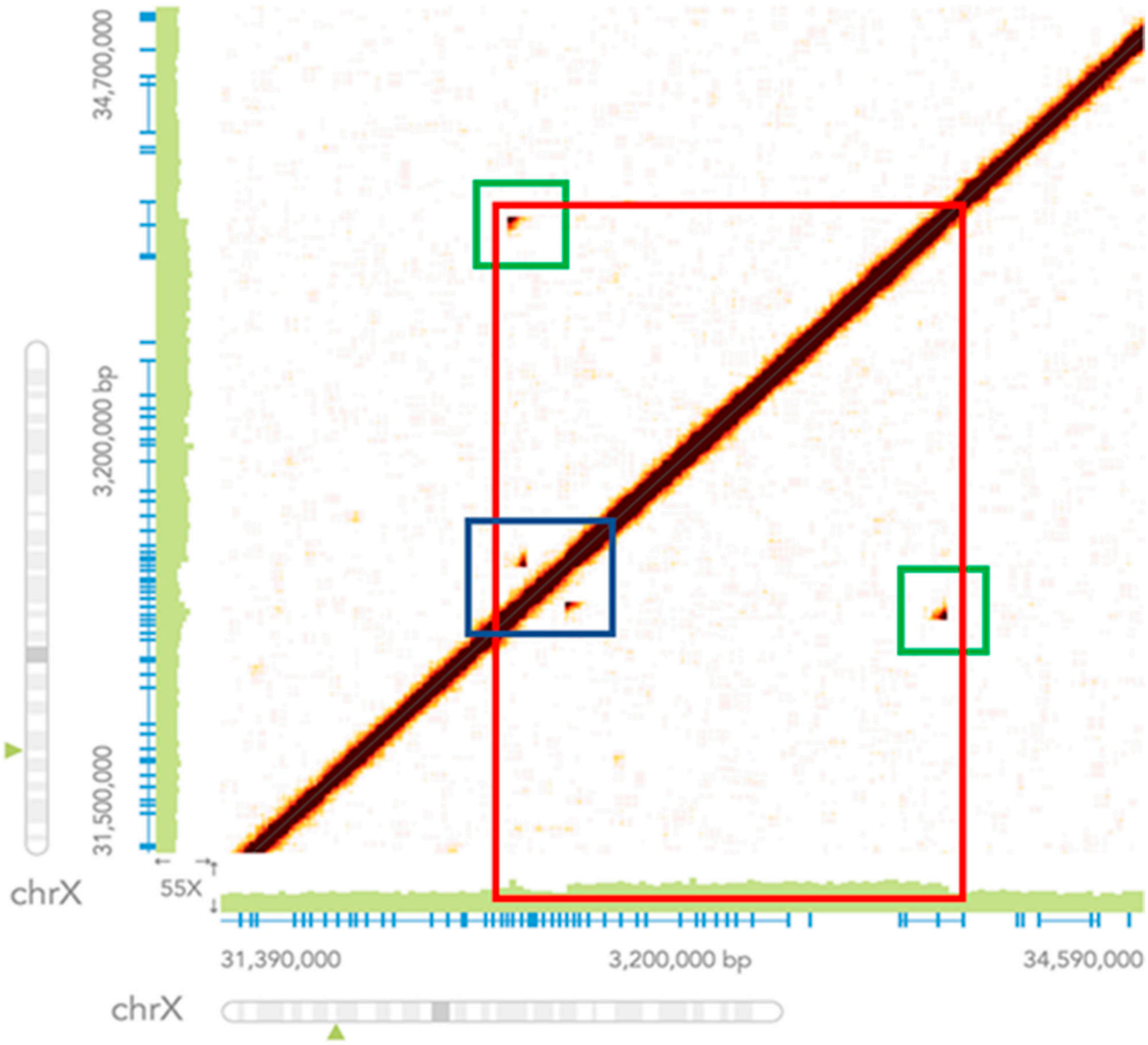

Supplement: Supplementary file 1 [file genes-12-00133-s001.pdf]
